# Clinical Applications of Immuno-PET in Lymphoma: A Systematic Review

**DOI:** 10.3390/cancers14143488

**Published:** 2022-07-18

**Authors:** Elizabeth Katherine Anna Triumbari, David Morland, Riccardo Laudicella, Matteo Bauckneht, Domenico Albano, Salvatore Annunziata

**Affiliations:** 1 Unità di Medicina Nucleare, TracerGLab, Dipartimento di Radiologia, Radioterapia ed Ematologia, Fondazione Policlinico Universitario A. Gemelli, IRCCS, 00168 Rome, Italy; elizabethkatherineanna.triumbari@guest.policlinicogemelli.it (E.K.A.T.); david.morland@wanadoo.fr (D.M.); salvatore.annunziata@policlinicogemelli.it (S.A.); 2 Service de Médecine nucléaire, Institut Godinot, 51100 Reims, France; 3 Laboratoire de Biophysique, UFR de Médecine, Université de Reims Champagne-Ardenne, 51100 Reims, France; 4 CReSTIC (Centre de Recherche en Sciences et Technologies de l’Informationet de la Communication), EA 3804, Université de Reims Champagne-Ardenne, 51100 Reims, France; 5 Department of Biomedical and Dental Sciences and Morphofunctional Imaging, University of Messina, via Consolare Valeria n1, 98125 Messina, Italy; 6 Department of Health Sciences (DISSAL), University of Genova, 16132 Genova, Italy; matteo.bauckneht@hsanmartino.it; 7 Nuclear Medicine, IRCCS Ospedale Policlinico San Martino, 16132 Genova, Italy; 8 Nuclear Medicine, ASST SpedaliCivili Brescia, 25122 Brescia, Italy; doalba87@libero.it; 9 Nuclear Medicine Department, University of Brescia, 25122 Brescia, Italy

**Keywords:** immuno-PET, lymphoma, systematic review, human, ^89^Zr, ^68^Ga-pentixafor

## Abstract

**Simple Summary:**

A systematic review of the published literature was performed to assess current clinicalapplications of immuno-PET in patients diagnosed with any histological type of lymphoma. The initial search yielded 1407 articles from PubMed/Medline and Scopus databases, but only 2 articles were found to comply with the inclusion criteria and 2 more were found during the cross-reference check. All four articles were deemed of sufficient methodological quality according to the QUADAS-2 assessment and were included in the review. Among the four included articles, three described the use of ^89^Zr-labelled antibodies targeting CD20+ relapsed/refractory B-cell lymphomas and one concerned the use of ^68^Ga-labelled mAb targeting CXCR4 in patients with non-Hodgkin lymphomas. Very limited literature data are currently available on the use of iPET in patients with lymphoma. However, iPET may represent a useful tool to non-invasively visualize the heterogeneous individual immunological environment, thus potentially guiding treatment-planning in lymphoma patients, and hence deserves further exploitation.

**Abstract:**

**Objective:** Immuno-positron emission tomography (iPET) combines the sensitivity of the PET imaging technique and the targeting specificity of radio-labelled monoclonal antibodies (mAb). Its first clinical applications in humans were described in the late 1990s, and several pathologies have benefitted from this molecular imaging modality since then. Our scope was to assess current clinical applications of immuno-PET in patients with lymphoma. Therefore, a systematic review of the published literature was performed. **Methods:** PubMed/Medline and Scopus databases were independently searched by two nuclear medicine physicians, to identify studies describing the clinical use of immuno-PET in patients with lymphoma. Methodological quality of the included articles was assessed by using the Quality Assessment of Diagnostic Accuracy Studies criteria. The studies were then analyzed concerning the molecular target of interest. **Results:** The initial search yielded 1407 articles. After elimination of duplicates, 1339 titles/abstracts were evaluated. Only two articles were found to comply with the inclusion criteria and two more were found during the cross-reference check. Among the four included articles, three described the use of ^89^Zr-labelled antibodies targeting CD20+ relapsed/refractory B-cell lymphomas and one concerned the use of ^68^Ga-labelled mAb targeting CXCR4 in patients with non-Hodgkin lymphomas. **Conclusions:** Very limited literature data are currently available on the clinical use of iPET in patients with lymphoma. This technique is encountering obstacles in its wider use, possibly because of the need of specific facilities, unfavorable dosimetry, and unclear correlation of immuno-tracer biodistribution with patients’ clinical and tumors’ molecular characteristics. However, iPET may represent a useful tool to non-invasively visualize the heterogenous individual immunological environment, thus potentially guiding treatment-planning in lymphoma patients, and hence deserves further exploitation.

## 1. Introduction

Immuno-positron emission tomography (iPET) is a whole-body molecular imaging technique that combines the high sensitivity, resolution, and quantitative ability of PET imaging to the specific binding properties of monoclonal antibodies (mAb) [1].

The understanding of how immunomodulation can engage patients’ immune system response to treat their cancer, with an improvement in response rates and overall survival, has progressively led to notable achievements in research and agencies’ approval of several immunotherapeutic agents, with CAR-T cell therapies being among the last, not the least, approved classes. Consequently, over time, clinicians, radiologists and nuclear medicine physicians came across new challenges, including the understanding of immune-related adverse effects (e.g., sarcoidosis), the correlation between patients’ and tumors’ characteristics (e.g., genomics, immunophenotypes) and prognosis, the interpretation of patients’ imaging (e.g., pseudo-progression cases) and the perception of [^18^F]FDG PET/CT limitations in assessing treatment response in patients undergoing immunotherapy [2,3]. In the era of personalized medicine and fast technological advancement, many disciplines discovered an interest in the characterization of tumors’ immunological environment [4,5,6], also to meet the well-established need for dose and timing optimization of treatment regimens [3]. Hence, the idea of targeting immune cells’ surface markers using radionuclide-labelled antibodies or their fractions for imaging and treatment purposes has been recently raised.

Hematological malignancies turned out to be appropriateground for radio-immuno-imaging and -therapy. The first clinical experiences were described about four decades ago, with preclinical and, more recently, clinical studies employing [^90^Y]ibritumomab tiuxetan [7] (Zevalin; IDEC Pharmaceuticals) and [^131^I]tositumomab (Bexxar; Corixa Corp.)to treat cancer with radioimmunotherapy (RIT) and performing pre-treatment imaging and dosimetry evaluations with ^131^I- and ^111^In-labelled antibodies on single photon emission computed tomography (SPECT) cameras [8]. The spread of more sensitive and accurate PET and PET/CT imaging systems in the early 2000s led to a shift of research investments towards the identification of PET radioisotopes, replacingγ-emitting radionuclides used in pre-RIT SPECT studies [9]. Only a few were selected and are currently under investigation (i.e., ^89^Zr, ^64^Cu, ^124^I, ^68^Ga), limiting the field of iPET application [10]. However, iPET can potentially quantify molecular interactions and simulate subsequent antibody-based therapies. Therefore, it remains of high interest in the field of immuno-oncology. Among others, hematological malignanciesare still steady targets both for radio-immuno-imaging and -therapy.

The scope of this systematic review was to gather all published information regarding the clinical application of iPET in patients with lymphoma, to describe the state of the art and understand its future direction.

## 2. Methods

For our purpose, we performed a systematic review of the published literature according to the standards of the PRISMA-P statement [11]. An electronic search of PubMed and Scopus databases was independently performed by two authors (E.K.A.T. and S.A.) to identify published manuscripts evaluating the clinical use of iPET in patients with lymphoma. The search strategy was built using synonyms for “PET”, “immuno-PET”, “labelled antibody” and “lymphoma” and was last updated on 22 March 2022. Articles were only included if they described the application of iPET on human patients diagnosed with any histological type of lymphoma, in a real clinical setting.

Duplicates, book chapters, review articles, case reports, congress communications, preclinical studies, off-topic articles, and articles not available in English were excluded during the initial assessment. Full texts of the potentially eligible studies were then retrieved for further evaluation. A cross-reference check was also performed to identify any additional studies to be included.

Two authors (E.K.A.T. and S.A.) independently assessed the methodological quality of the included articles using the standardized protocol provided by the Quality Assessment of Diagnostic Accuracy Studies (QUADAS-2) [12]. Any disagreement was solved by consensus. The QUADAS-2 scores of its four key domains (patient selection, index test, reference standard, flow and timing), evaluated with regards to the risk of bias and methodological applicability, were recorded and tabulated for all included studies, and a summary report was constructed. 

Year of publication, first author, country of origin, study design, aims, study population and population’s characteristics, employed tracer, molecular target, study phases, imaging protocoland main findings were extracted for each article.

Studies reporting data on the use of the same molecular target and/or immuno-tracer were analyzed altogether to obtain a summary result, when possible.

## 3. Results

**Figure 1:**
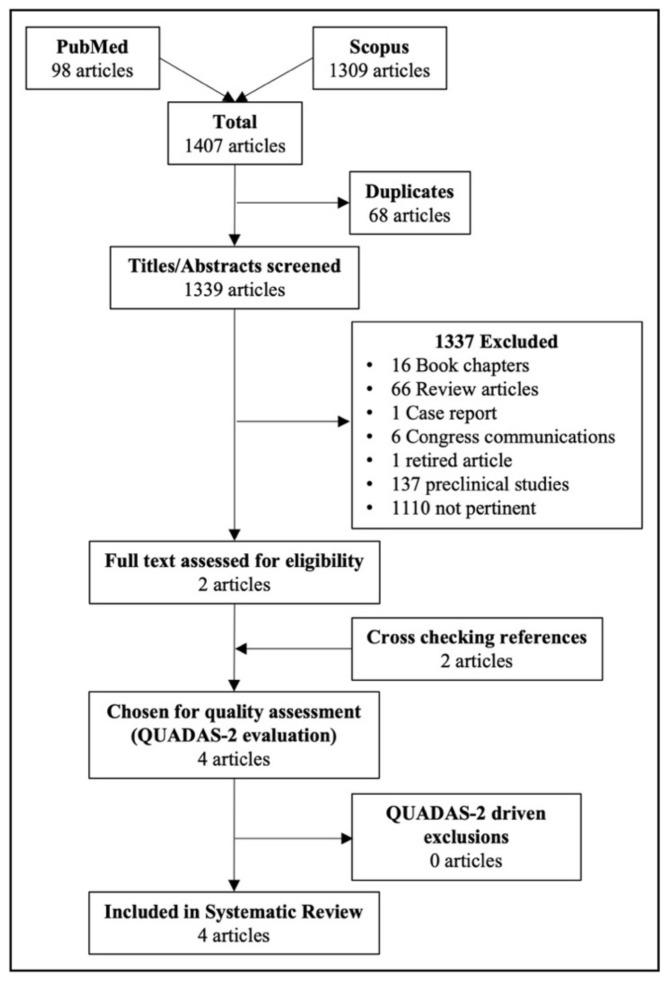
Flowchart of the literature search and article selection.

This systematic literature search initially yielded a total of 1407 articles (PubMed: 98 articles; Scopus: 1309 articles). Once the 68 duplicates were excluded, 1339 titles/abstracts were screened. More than 80% of the articles (1110/1339) did not correspond to the purpose of the study, mainly dealing with radioimmunotherapy and SPECT imaging modality (274/1110, 25%) or in-vitro immunological studies (383/1110, 35%). Only two articles met the inclusion criteria [13,14]. Full-text versions were retrieved and evaluated. Two additional studies were identified during the cross-reference check and deemed eligible [15,16]. Therefore, four articles were finally selected for inclusion and further assessment (Figure 1).

Results from the QUADAS-2 assessment are shown in Table 1. 

Withan unclear risk of bias only for patient selection and no applicability concerns in all evaluated key domains, all four studies were deemed of sufficient methodologic quality.

All studies were single-centered, prospective, and conducted in Europe between 2012 and 2017 (Table 2). The number of patients included was low (range: 3–7).Overall, 19 of the 21 patients studied with iPET in the four studies were diagnosed with B-cell lymphomas.

Three articles described the application of immuno-PET in CD20+ B-cell lymphomas [13,14,15], while the fourth article dealt with CD184 (better known as CXCR4, chemokine receptor type 4, or fusin) targeting with [^68^Ga]pentixafor in patients with non-Hodgkin’s lymphoma (NHL) [16]. Articles were, therefore, grouped to be presented depending on the molecular target of interest.

### 3.1. CD20 Targeting

Three out of four articles had CD20 as the molecular target for iPET [13,14,15]. CD20 is a transmembrane protein and a marker for B cells. Among its several roles, CD20 guides the growth and differentiation of B cells into plasma cells. Its overexpression on neoplastic B-cell surfaces makes it an ideal specific therapeutic target for immunotherapy of B-cell malignancies [17]. CD20 extracellular portion is notably exploited by rituximab, an unlabeled human chimeric IgG1 kappa anti-CD20 mAb that is already part of first line treatment in NHL. As lymphoma cells are also known to be highly radiosensitive, the action of labeling immunotherapeutic agents with radioactive molecules certainly gives CD20 an enormous potentiality to be the means of a radioimmunotherapy (RIT) treatment that is specifically designed to kill B cells, with the normal cells recovering within 6 months. The most studied and employed RIT compounds targeting CD20 are a ^131^I-labelled murine anti-CD20 IgG2a lambda mAb(tositumumab, Bexxar^®^, GlaxoSmithKline, Brentford, UK; withdrawn in the US in 2014 and in EU in 2015) and a ^90^Y-labelled murine IgG1 mAb (ibritumomab) in conjunction with the chelator tiuxetan (Zevalin^®^, Spectrum Pharmaceuticals Inc., Henderson, NV, USA), still licensed in EU and administered in combination with a preload of unlabeled rituximab [8].

More recently, ibritumomab-tiuxetan (IT) has been labeled with a positron-emitting isotope (^89^Zr) and the first successful human PET imaging study in one NHL patient was described by Perk et al. [18], showing selective tracer uptake in all tumor lesions that had previously been identified by [^18^F]FDG PET/CT. Favorable biodistribution of [^89^Zr]IT suggested the patient’s eligibility for [^90^Y]ibritumomab-tiuxetan RIT.

Among the three articles included in this review, the study by Rizvi et al. used Perk et al.’s experience to design a prospective study using [^89^Zr]IT immuno-PET [15]. The aim was to evaluate if [^89^Zr]IT PET scouts could predict [^90^Y]IT biodistribution and dose-limiting organ during treatment, and if the co-injection of [^89^Zr]IT and [^90^Y]IT would alter [^89^Zr]IT biodistribution. The number of patients studied was the highest among the four articles included in this systematic review; it consisted of seven patients with relapsed B-cell NHL, scheduled for autologous stem cell transplantation. However, not all patients underwent the same imaging protocol, which consisted of a standard treatment with 250 mg/m^2^ of unlabeled rituximab at the timepoints (T) 0 and T0 + 7 days, followed by administration of 68 ± 11 MBq of [^89^Zr]IT (T0 + 8 days) with PET/CT imaging at 1-, 72- and 144-h post-injection and several blood samplings for dosimetry purposes. Stem cell transplantation was performed at T0 + 40 days. At T0 + 54 days, four out of seven patients (because of logistical, not better explained, issues encountered) received a co-injection of [^89^Zr]IT with 15 MBq (in 2/4 patients) or 30 MBq (in the other 2/4 patients) of [^90^Y]IT and were again scanned at 1-, 72- and 144-h post-injection and examined through blood sampling for dosimetry. VOIs were drawn over entire organs that could be distinguished from the background (lung, liver, spleen, kidneys) and were used to calculate activity, % of injected dose and SUV values, tumor activity and dosimetric calculations for both [^90^Y]IT and [^89^Zr]IT. Overall, data from six patients were analyzed. All subjects tolerated [^89^Zr]IT well, with no reported adverse reactions. The 1-h scans revealed no tracer uptake in tumor sites, while blood-pool activity was assessable in the heart, liver, spleen, bone marrow and kidneys, in agreement with the experience by Perk et al. [18]. A progressive decrease in blood pool and increase in tumoral lesions activity was constantly observed in the 72- and 144-h scans. Biodistribution of [^89^Zr]IT was not influenced by simultaneous [^90^Y]IT therapy. The absorbed organ doses estimated using an [^89^Zr]IT scout prior to [^90^Y]IT therapy and those estimated using a simultaneous administration of ^89^Zr-IT with ^90^Y-IT showed high correlation. Only moderate correlation was found when tumor dosimetry was compared. The authors explained such a result by arguing that the data were drawn from only two timepoints, tumor lesions were smaller than organ volumes, and VOIs were not drawn from co-registered CT. Another possible reason could be attributed to the unlabeled antibody preload, which is, although, part of the established treatment protocols. The organ with the highest absorbed dose was found to be the liver, with 1.36 ± 0.58 mGy/MBq, followed by the spleen. The liver is also the organ with the highest absorbed dose in patients undergoing autologous stem cell transplantation. In this study, its absorbed dose was 9% of the threshold for nonstochastic effects [19], implying that [^90^Y]IT therapy with the administered- or higher-doses is safe.

Following this study, Muylle et al. questioned the possible interference of unlabeled antibody preload with iPET [13]. In their work, the employed mAb was rituximab, labelled with ^90^Y for RIT, and with ^89^Zr for iPET. As with radiolabeled IT, their first experiences describe an administration schedule comprehensive of a preload of unlabeled mAb [20], with the rationale that excess cold circulating mAb prolongs the half-life of circulating radiolabeled mAb, prevents toxicity (especially myelosuppression) by hindering nonspecific binding to normal tissues, while also increasing RIT therapeutic index. This study aimed at evaluating the impact of an unlabeled rituximab preload on the biodistribution of [^89^Zr]rituximab for prediction of tumor targeting and radiation dose of subsequent [^90^Y]rituximab therapy. All five patients included in the study were diagnosed with progressive CD20+ B-cell lymphoma and underwent the same imaging protocol, which consisted of the following three phases: (1) a diagnostic/dosimetric study, with 111 MBq of [^89^Zr]rituximab PET/CT dynamic imaging (T0); (2) a repetition of the diagnostic/dosimetric study, with the addition of a standard preload (250 mg/m^2^) of unlabeled rituximab 1–3 h before [^89^Zr]rituximab dynamic PET/CT (T0 + 3 weeks); (3) a therapeutic phase, with [^90^Y]rituximab RIT (11.1–14.8 MBq/kg, depending on platelet count </> 150.000/mm^3^), preceded by an unlabeled rituximab standard preload (T0 + 4 weeks). Whole-body [^89^Zr]rituximab scans were performed at three timepoints after tracer administration, including 1, 72 and 144 h. The results showed that in the dosimetric phase with unlabeled rituximab preload administration, the calculated whole-body effective dose of both ^89^Zr- and [^90^Y]rituximab was similar in all patients. With no preload, dosimetry was higher than with a preload, due to a higher radiation dose to the spleen in the two patients of the cohort presenting with preserved circulating CD20+ B cells (as a consequence of only one or two prior treatment regimens). The other three patients, which instead presented with B-cell depletion, had no significant difference in the whole-body dose but presented with a consistently higher tumor [^89^Zr]rituximab uptake than after preload. With no preload, radiation doses to the bone marrow were higher (9–58%) than with a preload, while there was no significant difference in the radiation dose to the liver. Overall, the study demonstrated tumor-targeting impairment after administration of the standard preload of unlabeled rituximab in patients with B-cell depletion (due to prior treatments with rituximab-containing therapeutic regimens), which usually are those eligible for RIT.

[^89^Zr]rituximab iPET was also employed by Jauw and colleagues to assess its CD20 targeting performance in patients with relapsed/refractory diffuse large B-cell lymphoma (DLBCL) [14]. DLBCL patients receive rituximab treatment within first-line therapeutic protocols. Early relapse (<1 year) and prior rituximab treatment have been associated with much worse outcomes, suggesting rituximab resistance [14]. Thus, in those patients, standard practice inclusion of rituximab in second-line therapies could be questioned. Assessment and quantification of target antigen expression via iPET would clear whether clinical benefit from mAb treatment is obtainable. With this rationale, six patients with biopsy-proven relapsed or refractory DLBCL after primary R-CHOP chemotherapy, and immunohistochemistry (IHC)-assessed CD20 positivity, were studied with 74 ± 10% MBq, 10 mg [^89^Zr]rituximab PET/CT. Injection of [^89^Zr]rituximab was performed on day 1 of the first cycle of second-line treatment, after the administration of a therapeutic dose of rituximab. The authors decided to follow the preload protocol, even though they were aware of the results described in the study by Muylle et al., to match with usual clinical therapeutic conditions as closely as possible. Patients’ blood samples were also collected for dosimetry. Tumor uptake was assessed at 1-, 72- and 144-h post-injection by qualitative and quantitative methods. I-PET scans were also compared to [^18^F]FDG restaging scans to confirm tumor localization. CD20 expression was assessed as present or absent and was semi-quantitatively evaluated as uniformly positive, heterogeneously positive, or absent. To correlate biopsy results with tumor uptake, patients were ranked based on CD20 expression levels. As previously reported by Muylle et al. [13], the administration of the immuno-tracer was well tolerated; only blood-pool uptake was evident in 1-h post-injection scans, while tumor uptake appeared and increased during following timepoint scans. Tumoral [^89^Zr]rituximab uptake and CD20 were positively concordant in four patients, negatively concordant in one patient, and discordant (CD20 negative, [^89^Zr]rituximab positive) in another patient. In this latter case, one possible explanation given was that discrepancy regarded an [^18^F]FDG-positive/ [^89^Zr]rituximab-negative biopsied lesion. Currently, IHC is the gold standard for CD20-expression determination, but heterogeneity in tumoral expression of the molecular target can cause false-negative results. A second hypothesis was that tumor uptake may not entirely depend on CD20 expression, but also on blood-tumor volume and non-target mediated binding. Overall, visual uptake intensity also correlated with CD20 tumor density at IHC, but the limited number of cases studied did not allow for the authors to understand whether rituximab iPET can predict a response to rituximab immunotherapy in patients with progressive DLBCL.

### 3.2. CXCR4 Targeting

The first description of CXCR4 targeting in patients with lymphoma was offered by Wester et al. in a proof-of-concept study that aimed at demonstrating the potential of [^68^Ga]pentixaforiPET for in-vivo quantification of CXCR4 expression for subsequent treatment strategies [16]. CXCR4 is also known as chemokine receptor type 4, fusin or CD184. It is expressed by T and B lymphocytes, monocytes, macrophages, neutrophils and eosinophils, hematopoietic and progenitor cells in the bone marrow. It is part of a large family of G-protein coupled receptors that mediates chemotaxis and cellular trafficking, and its specific function is to alter the expression of adhesion molecules and cell homing by binding with its ligand CXCL12 (stromal cell-derived factor 1). Such coupling has physiological roles in embryogenesis, hematopoiesis, and inflammation. Activation of the CXCR4-CXCL12 axis is also present in pathological conditions, such as vascular, autoimmune diseases and tumorigenesis. In the latter case, CXCR4 overexpression is a negative predictive factor, as it is associated with enhanced tumor growth and progression, tumor invasiveness and metastases [21]. Therefore, it has become a target for several immunotherapeutic and, more recently, RIT strategies [22,23]. While pre-clinical probes were successful, Wester’s group was the first to develop a radio-labeled compound targeting CXCR4 that was sensitive enough for quantification studies in humans, named [^68^Ga]pentixafor [16]. Pentixafor is a synthetic pentapeptide with high affinity for CXCR4. Corresponding pentixafor derivates can also be labelled with suitable therapeutic isotopes (beta- or alpha-emitters), making this tracer the first possible theranostic compound in radio-immuno-oncology.

In the article by Wester and colleagues, the pre-clinical phases described high species selectivity of the PET-ligand for human CXCR4, direct correlation of cellular accumulation of [^68^Ga]pentixafor with cellular CXCR4-expression levels and colocalization of CXCR4 and Ki67 expression patterns in high CXCR4 expressing tumors [16]. The clinical phase of the study included four patients, one of which was affected by multiple myeloma and not directly of interest for our review.Patient n.1 had CD30+ aggressive T-cell lymphoma and metachronous non-small-cell lung cancer; patient n.2 had relapsed DLBCL; patient n.3 was affected by chronic lymphocytic leukemia and suspect transformation into aggressive B-cell lymphoma.

The imaging protocol consisted of a standard [^18^F]FDG PET/CT and, on the next day, a PET/CT study performed after 50 min post-injection of 200 MBq of [^68^Ga]pentixafor. Normal organs and background tissues showed only little to moderate uptake, with an excellent lesion to background tissue uptake contrast ratio (one mild and expected exception represented by the bone marrow). The administration of [^68^Ga]pentixafor was well tolerated in the absence of any significant changes in vital parameters at 3 and 24 h post-injection. In addition, a dosimetric study revealed lower organ absorbed doses and a lower total effective dose of [^68^Ga]pentixafor, compared to other ^68^Ga-labeled tracers.

In patient n.1, the biopsied lymphoma lesion showed uptake of both [^18^F]FDG and [^68^Ga]pentixafor, while the lung tumor showed only scant [^68^Ga]pentixafor uptake. IHC confirmed no CXCR4 lung tumor-expression. Interestingly, some other lesions presented with high [^18^F]FDG activity and absent or faint [^68^Ga]pentixafor uptake, deemed to be due to lung cancer. Moreover, lesions showing uptake of both tracers presented heterogeneous uptake on a voxel-by-voxel basis. Excellent tumor uptake of [^68^Ga]pentixafor was also present in tumor lesions of patients n.2 and n.3 (with even higher quantitative measures compared to [^18^F]FDG).

Even if in a very small number of patients, this proof-of-concept study suggests that [^68^Ga]pentixafor is a highly selective and specific method for the in vivo quantification of CXCR4 expression and can be of particular value for novel CXCXR4 targeted therapies, which are currently under evaluation.

## 4. Discussion

Intrinsically, medicine has the constant objective to tailor the course of every patient’s clinical history through personalization of the available treatments. For each patient, each tumor is uniquely characterized by molecular traits that cannot be revealed through conventional imaging or coarse functional radiotracers, such as [^18^F]FDG. Immuno-PET, instead, is an ideal method to help with visualizing the heterogenous individual immunological environment via the radiotracer’s distribution in target lesions and healthy organs. It can guide consolidated immunotherapeutic strategies by ensuring the presence of adequate amounts of molecular targets for an effective immunotherapy [14], therefore predicting treatment outcomes; it can also simulate the biodistribution of radio-labelled antibodies to better select patients for receptor-targeted therapy and, through quantification, allow dosimetry as a prelude for RIT, defining patients at higher risk for RIT toxicity [20,24].

In case of hematological malignancies, RIT toxicity is predominantly hematological and depends on several factors, such as bone marrow reserve, absorbed dose and infiltration. Size of the spleen, number of circulating B-cells and tumor burden are among the factors influencing inter-patient variabilities in the radiotracer’s biodistribution and consequent bone marrow absorbed dose. One of the currently used strategies to avoid such toxicity is to administer patients with a preload of unlabeled mAb [8] before the injection of the radiopharmaceutical, which is thought to clear the peripheral blood of circulating B-cells and enhance targeting of tumoral B-cells within lymphoma lesions with the labelled antibody. However, the first study to investigate the consequences of this preload with iPET was by Muylle and colleagues [13], with the demonstration of an actual modification of tumor-targeting after cold mAb preload in patients that are usually eligible for RIT. Therefore, new studies on larger cohorts would be welcomed to define whether the standard amount of unlabeled mAb should be adjusted. Compared to [^90^Y]ibritumomab tiuxetan and [^131^I]tositumomab, radiolabeled rituximab is the only anti-CD20 mAb possibly allowing recruitment of B-cell lymphoma patients with bone marrow involvement, prior external beam radiation therapy of >25% of active marrow or prior autologous stem cell transplant [13,20].

Another way to limit RIT toxicity is to study the biodistribution of radiolabeled mAb and, specifically, discover dose-limiting organs with dosimetry studies [15]. Such a task is not properly achievable in the limiting frame of SPECT imaging. Namely, SPECT quantification can often over/under-estimate activity concentrations and impair dosimetry calculations. PET, instead, has proven high quantification abilities in preclinical immuno-imaging studies [18] and human oncological studies [24], overcoming other intrinsic limitations of SPECT imaging (e.g., spatial resolution). As a demonstration, in the study by Rizvi et al. [15], absorbed doses to the spleen were lower than those previously estimated using [^111^In]IT [25,26]. Such a finding was interesting, as EANM guidelines had already reported that dosimetry studies performed with [^111^In]IT in preclinical settings before [^90^Y]IT registration showed that currently available dose calculations could not efficiently predict therapeutic efficacy or toxicity of [^90^Y]IT treatments. Therefore, these dosimetry studies are currently not compulsory in the EU and immuno-SPECT imaging is not performed for dosimetric purposes, but to confirm expected biodistribution, as an additional safety measure before administering RIT [8].

A downside to PET quantification abilities is that, for iPET, the most used mAb-labelling isotope nowadays is ^89^Zr, with a long half-life (78.4 h) that makes it compatible with the time needed for a mAb to achieve optimal tumor-to-background ratio, even though this is with a higher effective dose than other conventional PET radionuclides (e.g.,^18^F). Several methodologies have been explored for organ [^89^Zr]-dosimetry assessment, and a harmonization proposal has already been made by Makris et al. [27].

Overall, the three articles describing CD20 targeting confirmed the high performance of iPET in assessing tumoral CD20 status and the high safety of RIT after iPET, while also unveiling differences in the biodistribution of the tracers depending on patients’ immunohistochemical and clinical characteristics, including previously undergone therapeutic schemes. This aspect was also underlined by the only article targeting CXCR4 [16], which suggested that the associated use of [^18^F]FDG and [^68^Ga]pentixafor could provide complementary information on tumor biology. A wider use of iPET is advocated in order to obtain better quantification studies and dosimetric models to be applied to more extensive populations.

Indeed, new advancements in technology and the spread of artificial intelligence (AI) applications to imaging have dramatically enhanced the ability to outline the heterogeneity of immune subsets and explore their functional state [3]. Total-body PET/CT systems will probably soon take the scene, ensuring a higher utilization of long-half-life isotopes for iPET, as their higher sensitivity allows for a dose reduction and an overall better comprehension of total-body pharmacokinetics. These scanners will also help to better understand toxicology, preload-interference issues, inconsistencies about dose-limiting organs and, most probably, to discover new biomarkers [28,29].

Research in lymphoma has made impressive progress also on the pharmaceutical side. Many studies are currently in search of new targets, new monoclonal antibodies, and new ways to label them and use them for pre-treatment imaging applications. High interest is being demonstrated towards CD30 targeting. CD30 is already the target of brentuximab vedotin (BV), an antibody-drug conjugate that has been approved for relapsed/refractory Hodgkin’s Lymphoma and anaplastic large T-cell lymphomas, cutaneous T-cell lymphoma andCD30+ peripheral T-cell lymphomas. CD30 is an ideal target antigen for antibody-mediated drug delivery, as it is over-expressed in lymphoma cells and not in healthy tissues and is readily internalized upon binding with BV, which is, therefore, being evaluated in clinical trials in combination with chemotherapy and other mAb, such as the anti-PD-L1 pembrolizumab [30]. 89Zr-radiolabeling of BV has been performed to study a lung cancer murine model, but the authors of the study foresee further applications in lymphoma models [31]. Another study targeted CD30 througha [^89^Zr]desferrioxamine (DFO)-labeled CD30-specific AC-10 antibody, which seemed a sensitive PET agent with high tumor-to-normal-tissue contrast for CD30 expression measurementand a promising radiotracer for clinical translation in patients with various lymphomas [32]. The already cited anti-PD-L1 mAbpembrolizumab has also been radiolabeled with ^89^Zr and investigated in a preclinical pharmacokinetics and biodistribution study [33]. CD19 is another target of interest for imaging and therapy, but no positron-emitting isotope has yet been used to label it for PET applications.

Several other radiopharmaceuticals with theranostic potential in the field of hemato-oncology are confined mostly to the experimental preclinical phase. However, ongoing research is a mine for future applications of iPET in lymphomas, which we believe will be of high importance to overcome the main limitation of the standard [^18^F]FDG PET/CT, in terms of detection of metabolically inactive lymphoma cells [1].

A clear limitation of the present study was the surprisingly low number of articles describing clinical applications of iPET in lymphoma. Moreover, the histological type of lymphoma described in the four included articles was B-cell in 90% of the reported cases.The number of patients included in each study was also very low and the obtained results could, therefore, lack generalizability, even if presented in a high-quality setting. To increase the number of articles and broaden the discussion on iPET in lymphomas, we could have chosen to investigate preclinical studies or some subsections, but it would have deviated from the original purpose of this review.

## 5. Conclusions

Although very limited data are currently available in the literature regarding iPET in patients with lymphoma, coupling the high sensitivity and quantification capabilities of PET with the high specificity of antibody binding properties potentially represent a winningstrategy that is able to increase patients’ treatment tailoring. This imaging modality is a promising path leading to improvement in efficacy, reduction in toxicity and costs of immunotherapy.

## Figures and Tables

**Table 1:** QUADAS-2 methodological quality assessment.

First Author, Year [ref]	Risk of Bias				Applicability Concerns		
	Patient Selection	Index Test	Reference Standard	Flow and Timing	Patient Selection	Index Test	Reference Standard
Muylle, 2015 [13]	?	☺	☺	☺	☺	☺	☺
Jauw, 2017 [14]	?	☺	☺	☺	☺	☺	☺
Rizvi, 2012 [15]	?	☺	☺	☺	☺	☺	☺
Wester, 2015 [16]	?	☺	☺	☺	☺	☺	☺

☺ = Low-risk; ? = unclear risk.

**Table 2:** Main characteristics of the included studies.

Author, Year [ref]	Aims	Target Lymphoma	Patients	Disease stage	Molecular Target	Monoclonal Antibody	Study Phases	Main Findings
Muylle, 2015 [13]	To compare [^89^Zr]Rit distribution with and without preload of unlabelledRit to assess the impact of preloading with unlabelledRit on tumor targeting and radiation dose of subsequent RIT with [^90^Y]Rit	B-cell lymphomas (1 NLPHL, 4 FL)	5 M; stages: I bulky (*n =* 1), II bulky (*n =* 1), II (*n =* 2), IV (*n =* 1)	Relapsed disease, no bone marrow involvement; at least one prior treatment regimen (mean: 3, range 1–4). If on Rit, discontinued at least 6 months before	CD20	Rit	Diagnostic/dosimetric phase I with [^89^Zr]Rit PET (at 1, 72 and 144 h) + diagnostic/dosimetric phase II with standard preload of unlabeled Rit before [^89^Zr]Rit PET+ therapeutic phase with preload of unlabeled Rit before [^90^Y]Rit administration	Without preload, increased whole-body effective dose of ^90^Y- and [^89^Zr]Rit in patients with preserved circulating CD20+ B cells compared to with preload; no difference between preload and no preload in whole-body effective dose among patients with B-cell depletion, although they consistently had higher tumor uptake in the phase with preload; radiation dose to bone marrow was higher with no preload
Jauw, 2017 [14]	Performance of [^89^Zr]Rit as an imaging biomarker to assess CD20 targeting before therapy with Rit	Biopsy proven DLBCL with no CNS involvement; histopathological assessment of CD20	6 (4M,2F); stages: IE (*n =* 1), IIA (*n =* 1), IIIA (*n =* 1), IIIB (*n =* 2), IVA (*n =* 1)	Relapsed or refractory DLBCL after first line R-CHOP therapy, before R-DHAP second-line therapy	CD20	Rit	[^18^F]FDG PET for relapse assessment; biopsy to prove relapse; IHC to rate CD20 positivity; [^89^Zr]Rit PET after therapeutic dose of Rit, with scans on day 0, 3 and 6	Tumor uptake and CD20expression wereconcordant in 5/6 patients; overall positive correlation
Rizvi, 2012 [15]	To assess [^90^Y]IT biodistribution and radiation dosimetry in humans using [^89^Zr]IT PET; evaluate if pre-therapy [^89^Zr]IT scout scans can predict [^90^Y]IT distribution during therapy; to predict dose-limiting organ during therapy	Relapsed/refractory CD20+ B cell lymphoma, not qualifiable for standard autologous stem cell transplantation; age < 66 years old	7 (4M, 3F)	Relapsed patients scheduled for autologous stem cell transplantation after R-CHOP in I line, R-DHAP, R-VIM and R-DHAP in II line; no partial remission at [^18^F]FDG PET	CD20	IT	Preload of unlabeled Rit before [^89^Zr]IT PET (at 1, 72 and 144 h, with dosimetric study) + stem cell transplantation + co-injection of [^89^Zr]IT and [^90^Y]IT in 4 patients (with[^89^Zr]PET at 1, 72 and 144 h, with dosimetric study)	High [^89^Zr]IT-image-based correlation between predicted pre-therapy and therapy organ absorbed doses; [^89^Zr]IT biodistribution is not influenced by simultaneous therapy with [^90^Y]IT; [^89^Zr]IT scout scans can predict biodistribution and dose-limiting organ during treatment; the dose-limiting organ in patients undergoing stem cell transplantation is the liver
Wester, 2015 [16]	First clinical application of [^68^Ga]CXCR4 targeted molecular imaging to human lymphoproliferative diseases	CD30+ aggressive T-cell lymphoma and metachronous NSCLC (*n =* 1); relapsed DLBCL (*n =* 1); chronic lymphocytic leukemia and suspected transformation into aggressive B cell lymphoma (*n =* 1)	3	Progressive disease (relapsed/refractory to first-line R-CHOP), before second-line treatment with rit+cisplatin-based chemotherapy	CXCR4	Pentixafor	Preclinical in vitro and in mice model study, pentixafor and [^18^F]FDG PET inpatientsin two consecutive days	[^68^Ga]pentixafor is a highly species selective PET-ligand for human CXCR4 and specific method for in vivo quantification of CXCR4 expression; CXCR4 expression correlates with cellular [68Ga]pentixafor uptake; excellent lesion to background tissue uptake contrast ratio (mild exception: bone marrow); favorable dosimetry

IT: Ibritumumabtiuxetan; Rit: rituximab; RIT: radioimmunotherapy; R-CHOP: rituximab, cyclophosphamide, jydroxydaunorubicin hydrochloride, oncovin, prednisone; R-DHAP: rituximab dexamethasone, cytarabine, cysplatin; R-VIM: rituximab, etoposide, ifosfamide, mitoxantrone; DLBCL: diffuse large B cell lymphoma; CNS: central nervous system; IHC: immunohistochemistry; NSCLC: non-small cell lung cancer.
